# Immigration status-related exclusive e-cigarette use and cannabis use and their dual use disparities associated with mental health disorder symptoms

**DOI:** 10.1016/j.drugalcdep.2024.111083

**Published:** 2024-01-06

**Authors:** David Adzrago, Saanie Sulley, Faustine Williams

**Affiliations:** a National Healthy Start Association, Washington, DC, USA; b Division of Intramural Research, National Institute on Minority Health and Health Disparities, National Institutes of Health, Bethesda, MD, USA

**Keywords:** E-cigarette use, Cannabis use, Tobacco use, Substance use, Mental health disorder, Immigration, Sexual and gender identity, Minority, Disparities

## Abstract

**Introduction:**

E-cigarette and cannabis use has been linked to various health risks, including respiratory and cardiovascular conditions. Yet, extant knowledge about the risk factors for exclusive and dual use of e-cigarettes and cannabis is limited, especially among immigrants. We examined exclusive e-cigarette and cannabis use and their dual use associated with mental health disorders among immigrants and U.S.-born.

**Methods:**

We analyzed national cross-sectional data collected between May 13, 2021, and January 9, 2022, among adults aged ≥18 years (n= 4766) living in U.S. Multinomial logistic regression analyses were conducted to model the associations of exclusivity and dual-use (reference group= non-use) with anxiety/depression.

**Results:**

The dual-use prevalence was higher than exclusive e-cigarette and cannabis use, especially among U.S.-born (dual use= 14.79% vs. cannabis use= 13.53% vs. e-cigarette use= 7.11%) compared to immigrants (dual use= 8.23% vs. cannabis use= 5.03% vs. e-cigarette use= 6.31%). Immigrants had lower risks of exclusive cannabis and dual use compared to U.S.-born. Anxiety/depression was associated with higher risks of exclusive cannabis use and dual use across immigration status, but was associated with exclusive e-cigarette use among only immigrants. While effect sizes of dual-use associated with anxiety/depression were higher among U.S.-born, the effect sizes of exclusive e-cigarette and cannabis use associated with anxiety/depression were higher among immigrants.

**Conclusions:**

The findings revealed significant mental health risks for e-cigarette, cannabis, and their dual use among immigrants and U.S.-born, especially among U.S.-born. These findings highlight the need for public health research and interventions to consider immigration status-related disparities in substance use.

## 1. Introduction

The United States (U.S.) is currently witnessing a profound shift in the patterns of prevalent substance use, primarily triggered by the rise of electronic cigarettes (known as e-cigarettes) and the progressive legalization of cannabis. E-cigarettes, commonly touted as a less harmful substitute to conventional tobacco products, have experienced a rapid rise in popularity, especially among younger age groups (Golan et al., 2023; Lewis et al., 2022; Short and Cole, 2021). Their marketing often highlights the perceived reduced risk, creating a narrative that leads to an increase in adoption among populations previously hesitant about tobacco usage (Do et al., 2022; Mantey et al., 2016; Ozga et al., 2023; Stanton et al., 2022; Zheng and Lin, 2023). Concurrently, the decriminalization and legalization of cannabis in numerous states may have aided in an uptick in the use of e-cigarettes and cannabis (Adhikari et al., 2021; Bhatia et al., 2022; Meng et al., 2022; Nicksic et al., 2020; Veligati et al., 2020). While these substances are often consumed independently, an emerging trend of dual use, defined as the concurrent use of e-cigarettes and cannabis, has been observed (Islam et al., 2023; Mattingly et al., 2023; Roberts et al., 2022; Williams et al., 2023). The increase in both e-cigarettes and cannabis may also be attributed to the use of e-cigarette products to deliver or administer cannabis (Chadi et al., 2020; Fataar and Hammond, 2019). This pattern of dual-use presents unique health risks that are becoming a significant public health concern (Azagba, 2018; Carlini et al., 2022; Davis et al., 2022). E-cigarette use, despite its perceived safety, has been linked to a range of health risks, including respiratory diseases and cardiovascular conditions (CDC, 2021; Cho et al., 2023; Marques et al., 2021). Furthermore, nicotine, the primary addictive substance in e-cigarettes, has been well-documented to have deleterious effects on cardiovascular health, including increased heart rate and blood pressure (CDC, 2023; HHS, 2016; Singh et al., 2020; Williams et al., 2013). Cannabis use, particularly at high doses, has also been associated with mental health disorders, cognitive impairment, and an increased risk of accidents (Albaugh et al., 2023; Brown et al., 2023; Cheng et al., 2023). The synergistic effects of these substances’ dual use could potentially exacerbate these health risks, leading to severe health outcomes. For instance, individuals who engage in dual-use may experience heightened respiratory issues due to inhaling e-cigarette vapor and cannabis smoke (Buckner et al., 2021). Additionally, the simultaneous use of these substances may lead to increased psychoactive effects of both substances and potentially higher risk of mental health disorders, such as anxiety and depression (SAMHSA, 2020b). Other areas of concern for dual use of these substances are the possible exacerbation of dependency issues, complicating the treatment process and negatively impacting the overall health outcomes.

Despite the potential health risks associated with the dual use of these substances, research in this area remains sparse. Most existing studies focus on using e-cigarettes (Kim et al., 2022; O’Brien et al., 2021; Stallings-Smith and Ballantyne, 2019) or cannabis (Robinson et al., 2022; Schlossarek et al., 2016; Van der Steur et al., 2020) in isolation, thereby overlooking the unique risks associated with their combined usage. This one-sided focus inadvertently leaves a significant gap in our understanding of the cumulative effects of these substances on an individual’s health, limiting our ability to respond effectively to this growing public health concern. Although some few emerging studies (Jacobs et al., 2023; Jones et al., 2023; Mattingly et al., 2022; McClure et al., 2023; Reboussin et al., 2021; Smith et al., 2022) have examined dual use of e-cigarettes and cannabis, there is a deficit of research investigating the risk factors for this dual use behavior based immigration status, especially when considering population subgroup-related risk factors such as mental health disorders, patterns of substance use, and sociodemographic differences.

In the general population, mental health disorder symptoms (e.g., anxiety, depression) are well documented risk factors for substance use (e.g., e-cigarette use, cannabis use, alcohol use), including dual and poly substance use (Conway et al., 2017; Duan et al., 2022; Kondracki et al., 2022; Lewis et al., 2022; Spears et al., 2019, 2020; Thrul et al., 2020). Individuals with mental health disorder symptoms are more likely to use substances such as e-cigarettes, cannabis, or their combination (Conway et al., 2017; Duan et al., 2022; Kondracki et al., 2022; Lewis et al., 2022; Spears et al., 2019, 2020; Thrul et al., 2020). However, the key factors such as underlying associations of the established patterns of substance use mental health disorder symptoms and sociodemographic differences have not been studied in the immigrant (i.e., individuals not born in the U.S.) and U.S.-born (i.e., individuals born in the U.S.) populations. Immigration status is a significant social determinant of health, especially in the U.S. where the highest number of immigrants worldwide live (Castañeda et al., 2015; DeFries et al., 2022; Kagotho et al., 2020; Martinez et al., 2015). Immigrants are also one of the most vulnerable, disadvantaged, and minority groups that experience greater risks of poor health and substance use disorder complications (DeFries et al., 2022; Grace et al., 2018; Kagotho et al., 2020). This gap in the literature is particularly pronounced for immigrant populations, who are often understudied in substance use research despite potentially facing unique risks and challenges (Bustamante et al., 2021). Despite immigrants’ significant presence in the U.S. (Budiman, 2020), they often remain understudied, potentially due to language barriers, cultural nuances, or logistical issues related to data collection (Berry, 2006; Klein et al., 2020; Lee et al., 2013). This lack of attention is concerning, as immigrant populations may face unique risks and challenges associated with substance use due to acculturation stress, socioeconomic inequalities, or limited access to healthcare services (Berry, 2006; Lee et al., 2013). The existing studies indicate that immigration status plays major roles in substance use because immigration stressors (e.g., legal status, forced migration, historical trauma, violence, family separation, and poverty) contribute to vulnerability and increased risk of substance use (DeFries et al., 2022; Marginean et al., 2023; Salas-Wright et al., 2014). However, none of the existing studies examined dual use of e-cigarette and cannabis, or their exclusivity with mental health disorder symptoms in immigrants and U.S.-born to identify the disparities in this behavior for personalized public health interventions.

Given the increasing prevalence of e-cigarette and cannabis use and the potential health risks associated with their dual use, it is crucial to expand the literature on and our understanding of the factors contributing to this dual-use behavior based on immigration status. This study aims to fill the gap in the literature by (1) estimating exclusive e-cigarette and cannabis use and their dual use by mental health disorder symptoms and sociodemographic characteristics based on immigration status, (2) the associations of exclusive e-cigarette and cannabis use and their dual use with immigration status, adjusting for mental health disorder symptoms and sociodemographic characteristics, and (3) the associations of exclusive e-cigarette and cannabis use and their dual use with mental health disorder symptoms, adjusting for sociodemographic characteristics, among immigrants and U.S.-born. Consequently, our findings will shed light on this understudied area and inform targeted interventions to mitigate the potential risks associated with dual-use behaviors.

## 2. Methods

### 2.1. Study design and participants

We analyzed national cross-sectional data that were collected as part of a study, *Understanding the Impact of the Novel Coronavirus (COVID-19) and Social Distancing on Physical and Psychosocial (Mental) Health and Chronic Diseases*, among adults aged 18 years or older living in the U.S. This survey was an anonymized, online or web-based survey that was conducted among a random sample of the U.S. adults. The participants’ recruitment, screening, enrolment in the study, and survey administration were conducted between May 13, 2021, and January 9, 2022, by Qualtrics LLC using their existing survey panels. Qualtrics used demographic characteristics of a theoretical cohort to randomly match eligible panel members and drew the sample, including US- and foreign-born (i.e., immigrant) adults, from the American Community Survey. Low income (<$30,000 annual household income) and rural adults were oversampled among US-born White, Black, and Hispanic and foreign-born population to ensure representativeness of the participants. Qualtrics compensated each participant with a $5–$10 gift card for completing the survey. The survey was developed in English and distributed to 10,000 participants in English with about 59.38% response rate representing 5938 surveys received by Qualtrics LLC. Information Management Services (Carlini et al.), Inc. was contracted to review and correct the de-identified data based on the survey completeness criteria (completed ≥80% of the 102 survey questions for not less than 5 minutes). IMS determined 5413 participants had accurately completed the surveys that formed the final sample for the study. Further details about this survey have been published elsewhere (Talham and Williams, 2023). We conducted a complete case analysis of 4766 participants out of the 5413 participants for this current analysis to ensure that we included only the participants with no missing data on our variables of interest. The National Institutes of Health’s Institutional Review Board determined the study as exempt (IRB #000308) on 12/23/2020. We used Strengthening the Reporting of Observational Studies in Epidemiology (STROBE) to guide the writing of this paper (Vandenbroucke et al., 2007).

### 2.2. Measures

#### 2.2.1. Outcome variable

Exclusive e-cigarette and cannabis use and dual use of e-cigarettes and cannabis were assessed with two questions: During the past month, how often did you (1) smoke e-cigarettes or use vaping products? and (2) use cannabis? The responses include 1= not at all, 2= once during the month, 3= several times during the month, 4= once a week, 5= several times a week, 6= every day or almost every day, and 7= several times a day. We dichotomized the responses into use (response options 2–7) and did not use (option 1). Next, we combined the responses into a single categorical variable to indicate non-use (if participants did not use both products), exclusive e-cigarette use (if participants used e-cigarettes but not cannabis), exclusive cannabis use (if participants used cannabis but not e-cigarettes), and dual-use (if participants used both products).

#### 2.2.2. Exposure variables

Immigration status and mental health disorder symptoms (anxiety/depression, Post-Traumatic Stress Disorder [PTSD], and loneliness) were the exposure variables. Immigration status was determined by asking the participants whether they were born in the U.S. (including all 50 states and the District of Columbia) or outside U.S. (not excluding Puerto Rico and other US territories). Those not born in the U.S. were referred to as immigrants, while being born in the U.S. was considered U.S.-born or non-immigrants.

Anxiety/depression symptoms were assessed with four survey questions based on the Patient Health Questionnaire-4 (PHQ-4) scale. The participants were asked how often they experienced anxiety and depression symptoms in the last two weeks. Specifically, they were asked if they have been disconcerted by (1) feeling nervous, anxious or on edge, (2) not being able to stop or control worrying, (3) feeling down, depressed, or hopeless, and (4) little interest or pleasure in doing things (Kroenke et al., 2009a; Löwe et al., 2010). The response options for each of the four questions include not at all = 0, several days = 1, more than half the days = 2, or nearly every day = 3, with a total PHQ-4 score ranging from 0–12. In this study, we analyzed the PHQ-4 cutoff points used to determine minimal/negative (score= 0–2), mild (score= 3–5), moderate (score= 6–8), and severe (score= 9–12) anxiety/depression symptoms(Kroenke et al., 2009b; Löwe et al., 2010).

PTSD, based on the Primary Care PTSD (PC-PTSD-5) screen for the Diagnostic and Statistical Manual of Mental Disorders 5th edition (DSM-5), involves five items used to identify probable PTSD in participants. The participants were first asked (to determine their eligibility for the five items) if they ever (yes/no) experienced any frightening, horrible, or traumatic events (e.g., accident/fire, war, environmental disaster, assault). Those who reported experiencing any of such events were further asked to respond (yes/no) to five items about their experiences in the past month: (1) Had nightmares about the event(s) or thought about the event(s) when you did not want to? (2) Tried hard not to think about the event(s) or went out of your way to avoid situations that reminded you of the event(s)? (3) Been constantly on guard, watchful, or easily startled? (4) Felt numb or detached from people, activities, or your surroundings? (5) Felt guilty or unable to stop blaming yourself or others for the event(s) or any problems the event(s) may have caused?(Prins et al., 2016). Participants who answered “yes” to three or more of the five questions were considered to have PTSD symptoms (Prins et al., 2016). Otherwise, the participants screened negative for PTSD. We further categorized the participants into three groups: (1) ineligible/unqualified for PC-PTSD-5, (2) eligible/qualified for PC-PTSD-5 but had no PTSD, and (3) eligible/qualified for PC-PTSD-5 and had PTSD.

Loneliness was measured with a 3-item UCLA Loneliness Scale (a short version) among the participants. The participants were asked about how often they (1) lack companionship, (2) feel left out, and (3) feel isolated from others (Hudiyana et al., 2022; Hughes et al., 2004; Russell, 1996). The possible answers to the three items include hardly ever= 1, some of the time= 2, and often= 3. The participants can only select one option per item. The total score for the three items ranges from 3 to 9, with higher scores indicating higher loneliness. We evaluated the reliability of the loneliness scale (UCLA Loneliness Scale - Short) using Cronbach alpha (α) and found strong internal consistency or reliability of the scale among U.S.-born (α= 0.88) and immigrants (α= 0.87).

#### 2.2.3. Covariates/confounders

Individual-level factors/variables were included in the analysis based on previous studies that established their significant associations with substance use (Barger et al., 2021; Compton et al., 2023; Garrison-Desany et al., 2023; Jun et al., 2019; SAMHSA, 2020a, 2022). These factors were age, gender identity (man, woman, non-binary, or transgender), sexual orientation (lesbian, gay, bisexual, or heterosexual), race/ethnicity (Black/African American, other [American Indian/-Alaskan Native, Pacific Islander, Asian, multi-racial], Hispanic/Latino, or White), level of education completed (less than high school, high school diploma or GED, some college/vocational or technical school, or college/higher education), and U.S. census region (Northeast, West, Midwest, and South). For this study and due to limited samples within groups, we dichotomized sexual orientation into heterosexual and sexual minority (lesbian, gay, and bisexual). We also included past-month alcohol use, which was determined with similar questions as those used for cannabis and e-cigarette use.

### 2.3. Statistical analysis

**Fig. 1.**
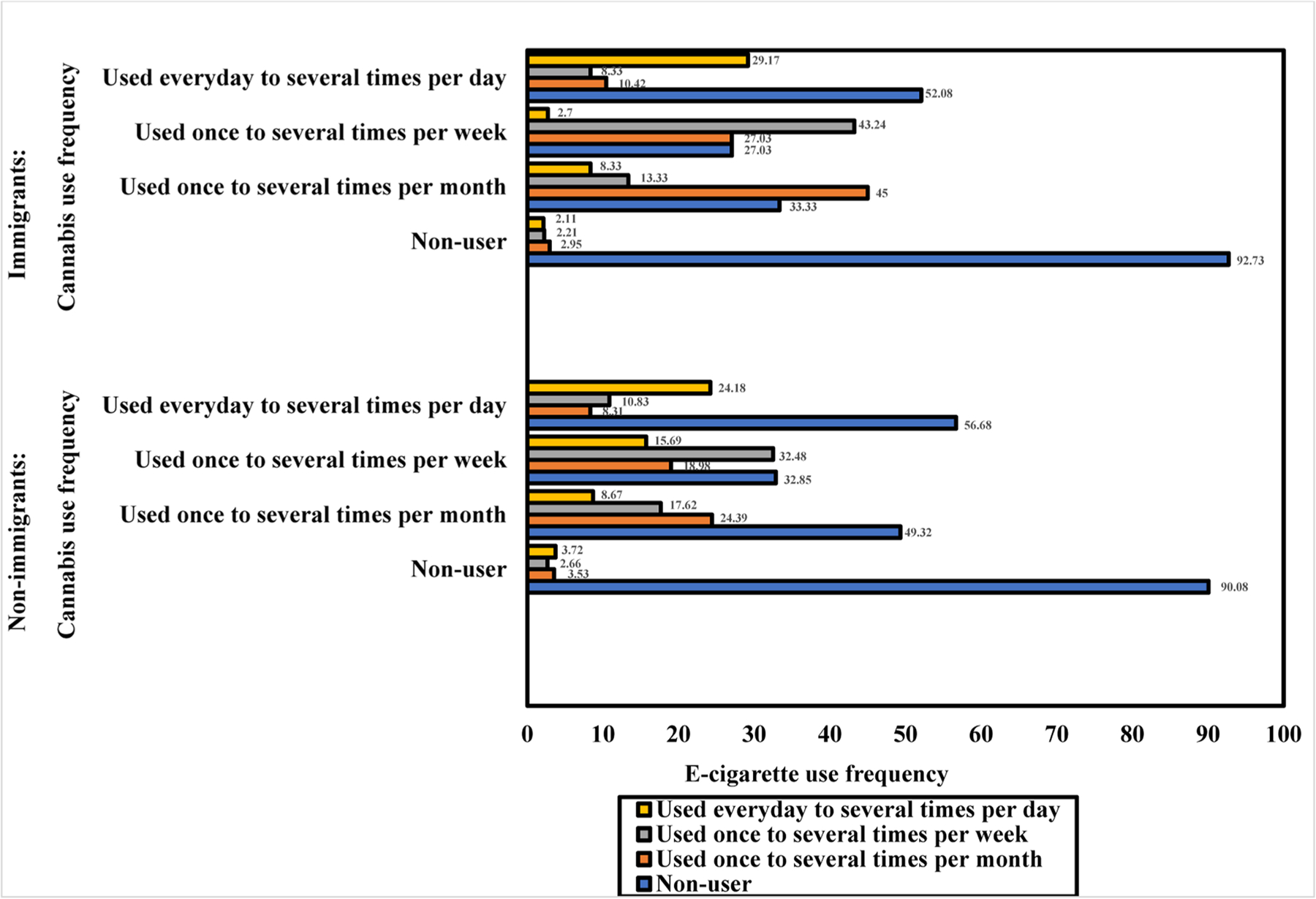
Prevalence of e-cigarette use frequency by cannabis use frequency stratified by immigration status among U.S. adults.

Before we combined e-cigarette and cannabis use, we estimated the intersection of their use frequencies stratified by immigration status (Fig. 1). Next, we conducted descriptive and bivariate analyses to determine the prevalence of exclusive e-cigarette and cannabis use and their dual use by sociodemographic characteristics, mental health disorder symptoms, and alcohol use based on immigration status (Table 1). The bivariate statistics were computed using Chi-Squared tests or analysis of variance (ANOVA) to determine group differences in the outcome variable. We conducted multinomial logistic regression analyses to model the associations of exclusive e-cigarette and cannabis use and their dual use (reference group= non-use) with mental health disorder symptoms, adjusting for sociodemographic characteristics and alcohol use, among immigrants and U.S.-born (Table 2.2). Before stratifying the logistic regression model by immigration status, we examined the association between the outcome variable and immigration status, adjusting for sociodemographic, mental health, and alcohol use characteristics (Table 2.1). We reported relative risk ratios (RRRs) with 95% confidence intervals (CIs) for the estimates. The statistical significance level was determined at p<0.05. Before conducting the multinomial logistic regression analyses, we evaluated the association between the predictors to determine their multicollinearity. The mean variance inflation factor (VIF) was 1.21, indicating no significant multicollinearity because the VIF is lower than VIF value of 10 to be considered serious multicollinearity. Analyses were conducted using STATA version 16.1.

## 3. Results

### 3.1. Descriptive characteristics of the participants by immigration status

The characteristics of the participants are presented by immigration status in Table 1. Of the 3672 U.S.-born, most of them were within the age 35–49 years (32.30%), identified as a woman (62.99%), heterosexual (89.79%), White American (49.51%), some college/vocational or technical school (35.78%), and resided in the U.S. South (46.16%). A significant proportion of the U.S.-born also experienced mild (22.88%), moderate (13.97%, and severe (12.83%) anxiety/depression symptoms. About 10.29% of them experienced PTSD symptoms. They had a mean loneliness score of 5.06 (2.06), and most of them engaged in alcohol use (60.32%) in the past month. Of the 1094 immigrants, most of them were 35–49 years (29.25%), identified as a woman (63.62%, heterosexual (87.75%), Latino/Hispanic (35.37%), had college or higher education (51.19%), and resided in the U.S. South (42.96%). A higher proportion of them experienced mild anxiety/depression symptoms (21.48%), followed by moderate (11.15%) and severe (8.96%) symptoms, respectively. About 8.32% experienced PTSD symptoms. A mean loneliness score of 4.79 (SD= 1.93) was reported among them. More than half of them engaged in alcohol use in the past month (51.74%).

### 3.2. Differences in the prevalence of exclusive e-cigarette and cannabis use and their dual use by immigration status

Among the U.S.-born (Fig. 1), most individuals who used e-cigarettes and used them once to several times per week also used cannabis once to several times per week (32.48%). The next groups were those who used both e-cigarettes and cannabis daily to several times per day (24.18%) or used both products once to several times per month (24.39%). For the distributions in the immigrants, the majority of those who used e-cigarettes and used them once to several times per month also used cannabis once to several times per month (45%) or week (27.03%). The next group of individuals who used e-cigarettes was those who used both products once to several times per week (43.24%). About 29.17% of them who used e-cigarettes daily to several times per day also used cannabis daily to several times per day.

Stratified by immigration status, Table 1 shows that the prevalence of e-cigarette and cannabis use varied significantly by the participants’ sociodemographic characteristics, mental health, and alcohol use. The prevalence of dual use of e-cigarettes and cannabis (14.79%) was higher than the prevalence of exclusive cannabis use (13.53%) and exclusive e-cigarette use (7.11%) among U.S.-born, while the prevalence of dual use (8.23%) was also higher than the exclusive e-cigarette use (6.31%) and exclusive cannabis use (5.03%) among immigrants. The prevalence of dual use of e-cigarettes and cannabis was most common than exclusive use within all the subgroups of U.S.-born and immigrants. For instance, within subgroups in U.S.-born and immigrants, majority of those who engaged in dual use identified as non-binary/transgender, sexual minority, Black/African American, had less than high school education, experienced severe anxiety/depression symptoms, had higher loneliness scores, and used alcohol in the past month. Most of the individuals who engaged in dual use among the U.S.-born group had similar statistically significant sociodemographic, mental health, and alcohol use characteristics as those in the immigrants, except based on age (U.S.-born aged 18–25 or 26–34 years vs. immigrants aged 18–25 years), U.S. census region (U.S.-born= U.S. West vs. immigrants= results did not significantly vary), and PTSD status (U.S.-born had PTSD vs. immigrants did not have PTSD).

### 3.3. Associations of e-cigarette and cannabis use with mental health, sociodemographic, and alcohol use factors

As shown in Table 2.1, immigrants (vs. U.S.-born) had significantly lower risks of engaging in exclusive cannabis use and dual use of e-cigarettes and cannabis (reference group: non-use), adjusting for sociodemographic, mental health, and alcohol use characteristics. The model fit information (Х^2^ (72, N= 4766) = 1668.09, p<0.001) for the model in Table 2.1 suggests that this model fits significantly better than a model without any predictors. Table 2.2 presents multinomial logistic regression models for e-cigarette and cannabis use (reference group: non-use), stratified by immigration status. The model fit information for U.S.-born (Х^2^ (69, n= 3672) = 1297.84, p<0.001) and immigrant (Х^2^ (69, n= 1094) = 324.64, p<0.001) samples in Table 2.2 indicates that these models significantly improved with addition of the predictors and covariates. In U.S.-born, individuals with mild, moderate, and severe anxiety depression symptoms (reference group: no symptoms) had significantly higher risks of engaging in exclusive cannabis use and dual use. Those who were ineligible for PTSD assessment (reference group: had no PTSD symptoms) had significantly lower risks of using cannabis exclusively. The results also showed risks associated with the covariates/controlled factors. Compared to individuals aged 18–25, those aged 50 years or older were significantly less likely to use e-cigarettes or dual-use e-cigarettes and cannabis exclusively. Those who identified as a woman had significantly lower risks of engaging in exclusive e-cigarette and cannabis use or dual use compared to those who identified as a man; non-binary/transgender/other had significantly lower risks of exclusive cannabis use. Sexual minority individuals (vs. heterosexual persons) had significantly higher risks of exclusive cannabis use. Black/African American individuals (vs. White American individuals) were significantly more likely to engage in exclusive cannabis use and dual use. Education was significantly associated with lower risks of engaging in dual use. College or higher education (vs. less than high school) significantly decreased the risks of an exclusive cannabis use. Residing in the U.S. Northeast, Midwest, or South was significantly associated with lower risks of a dual-use behavior compared to residing in the West. Those residing in Midwest and South also had significantly lower risks of exclusive cannabis use behavior. Alcohol use was significantly associated with exclusive e-cigarette and cannabis use and their dual use.

Among immigrants (Table 2.2), individuals with moderate anxiety/depression symptoms had significantly higher risks of engaging in exclusive e-cigarette use and dual use, while severe anxiety/depression symptoms were significantly associated with higher risks of exclusive e-cigarette and cannabis use or dual use. Those with PTSD symptoms were significantly more likely to engage in exclusive cannabis use. The following covariates or controlled factors were significantly associated with e-cigarette and cannabis use. Individuals aged 65 years or older had lower risks of engaging in exclusive e-cigarette and cannabis use and their dual use compared to those aged 18–25. Persons who identified as a woman (vs. a man) were less likely to engage in exclusive cannabis use behavior or dual-use behavior. Black/African American and other racial/ethnic groups had lower risks of exclusive use behavior compared to White American individuals. College or higher education (vs. less than high school) was associated with lower risks of exclusive e-cigarette use. The risks of engaging in an exclusive e-cigarette and cannabis use and their dual use were associated with alcohol use.

## 4. Discussion

Our study provides a comprehensive analysis that sheds light on the prevalence of e-cigarette and cannabis use behaviors among immigrant and U.S.-born populations. It also examines the correlations of these behaviors with mental health disorder symptoms, including anxiety/depression, PTSD, and loneliness. A key finding from our research is that the dual use of e-cigarettes and cannabis was more prevalent than their exclusive use across all subgroups in U.S.-born and immigrants, especially in U.S.-born subgroups, in our study. Specifically, the prevalence of dual use was higher in both immigrant and U.S.-born individuals who identified as non-binary/transgender, sexual minority, Black/African American, young adult, had less than high school education, experienced severe anxiety/depression symptoms, had higher loneliness scores, and used alcohol. These findings are consistent with the observations of other researchers, who found that individuals with lower and underserved socioeconomic and sociodemographic characteristics (e.g., young adults, people with less than high school education, sexual and gender minority persons, Black/African American individuals) and mental health disorder symptoms (e.g., anxiety/depression, stress) were more likely to engage in substance use, including e-cigarette and cannabis use, particularly their combinations (Adzrago et al., 2022; Clendennen et al., 2021, 2023; Conway et al., 2017; Duan et al., 2022; Kondracki et al., 2022; Lewis et al., 2022; Spears et al., 2019, 2020; Thrul et al., 2020). However, none of the aforementioned studies examined subgroup differences in dual use of e-cigarettes and cannabis within immigrant and U.S.-born populations to identify immigration status-related disparities in dual use behavior for tailored substance use interventions aimed at reducing substance use and its health consequences, especially the increased risks of dual use of substances. Our findings also revealed a shared vulnerability to dual substance use behavior among individuals with mental health disorder symptoms irrespective of immigration status, but with higher effect sizes among U.S.-born. This association between substance use and mental health underlines the importance of an integrated approach to tackling this issue, where mental health and significant social determinants of health (e.g., immigration status) considerations are treated as integral to substance use interventions (Castañeda et al., 2015; Conway et al., 2017; DeFries et al., 2022; Duan et al., 2022; Grace et al., 2018; Kagotho et al., 2020; Kondracki et al., 2022; Lewis et al., 2022; Martinez et al., 2015; Spears et al., 2019, 2020; Thrul et al., 2020). Furthermore, the widespread prevalence of dual use across immigrant and U.S.-born populations, particularly U.S.-born, indicates the need for personalized prevention strategies alongside those targeted toward specific high-risk groups.

Our research also found that immigrant populations, adjusting for sociodemographic factors, mental health status, and alcohol use characteristics, exhibited lower risks of engaging in exclusive cannabis use and dual use of e-cigarettes and cannabis. Immigrants, in general, are known to be less likely to engage in substance use or misuse behaviors than U.S.-born (Johnson et al., 2002; Salas-Wright et al., 2018). This finding further supports the premises of the “healthy immigrant effect,” which suggests that immigrants tend to have better health outcomes than their host country natives (Ru and Li, 2021; Salas-Wright et al., 2018). Immigrants’ substance use behavior may be due to fear or concerns of being involved in risky or illegal behaviors that have immigration consequences (e.g., deportation) (Vaughn et al., 2014). Nonetheless, little to no studies examined exclusive and dual use of cannabis and e-cigarettes among immigrants and U.S.-born. The unique cultural and immigration-rated experiences of immigrants may have different implications for their substance use behaviors. Thus, comparing the behavior of subgroups of immigrants and U.S.-born may provide detailed information about specific group differences for tailored substance use prevention interventions. The findings also underline the importance of a context-specific understanding of the diverse factors related to e-cigarette or cannabis use among immigrants and U.S.-born. Immigrants, depending on their cultural backgrounds, reasons for migration, and experiences post-migration, may have different attitudes toward substance use compared to U.S.-born or other demographic groups (Prado et al., 2009; Tran et al., 2010). These attitudes are likely shaped by a confluence of factors such as societal norms, personal experiences, and access to substances, which can substantially impact the patterns of substance use.

In relation to mental health, our findings showed that individuals with mild, moderate, and severe anxiety/depression symptoms had higher risks of engaging in exclusive cannabis and e-cigarette use and their dual use across immigrant and U.S.-born populations. These findings align with the study by Burke et al. and Chloe et al., which found positive associations of tobacco use with mood disorder, psychotic disorder, and anxiety disorder (Buckner et al., 2021; Chloe et al., 2023). They also found a positive association between cannabis use and these disorders (Buckner et al., 2021; Chloe et al., 2023). This consistency across studies underscores the complex interplay between substance use and mental health disorders and the need for integrated interventions that address both issues. It also indicates that mental health status may contribute to the observed heterogeneity in substance use behaviors within these groups. Our findings further revealed that while exclusive e-cigarette use behavior was not significantly associated with anxiety/depression symptoms among U.S.-born, this behavior was significantly associated with anxiety/depression symptoms among immigrants, suggesting unique immigration status-related disparities in substance use behavior and mental health for consideration.

The findings on the influence of mental health further revealed that while U.S.-born individuals who were ineligible for PTSD assessment were significantly less likely to exclusively use cannabis, immigrant individuals with PTSD symptoms were significantly more likely to exclusively use cannabis. However, exclusive e-cigarette use behavior and dual use behavior were not significantly associated with PTSD across immigrant and U.S.-born individuals. Similarly, neither exclusive nor dual use of e-cigarettes and cannabis was significantly associated with loneliness across immigrant and U.S.-born individuals. The findings suggest that while mental health disorder symptoms may be associated with substance use behavior, the associations may vary depending on specific substances, mental health disorder symptoms, and target populations. Thus, the substance use behavior and mental health disorder symptoms may be different or similar in immigrant and U.S.-born populations depending on the specific substances and mental health disorder symptoms. These findings also emphasize the importance of disaggregating data to delineate and identify specific health behaviors associated with specific mental health disorder symptoms within specific populations for tailored public health and clinical interventions in addressing health disparities, especially in minority populations (Diaz et al., 2021; Etowa et al., 2021; Kauh et al., 2021; Quint et al., 2021). Aggregated data on immigration status can mask or obscure health behavior disparities among subgroups (Choi et al., 2023; Etowa et al., 2021; Lee et al., 2022; Quint et al., 2021). Consequently, we found that some subgroups within U.S.-born and immigrants exhibited more noticeable disparities in substance use behaviors. For instance, while some groups had consistent substance use behavior in immigrant and U.S.-born populations, others had inconsistent behaviors. Similar to other research findings, we observed that non-Hispanic Black/African American individuals were more likely to engage in dual-use behaviors in the U.S.-born population, but no differences in such behavior in the immigrant population (Uddin et al., 2020). The findings highlight the need for more studies, especially longitudinal studies, to quantify changes in substance use behavior within the subgroups to enhance deeper understanding of the consistency of this behavior and mental health among immigrants and U.S.-born.

Consistent with the findings of other studies, we found that among U.S.-born, individuals identifying as a sexual minority and Black/African American had higher risks of exclusive cannabis use and dual use (Adzrago et al., 2021; Dyar et al., 2021; Swann et al., 2020). Among immigrants, sexual minority and Black/African American individuals had lower risks of exclusive e-cigarette use. This observation could be further explained by the findings of other studies that observed lower smoking, e-cigarette use, and substance use prevalence among immigrants (Bosdriesz et al., 2013; Salas-Wright et al., 2014; Wang et al., 2016). These findings emphasize the need to evaluate health behaviors and outcomes within specific population subgroups to better delineate the related disparities. While immigrants generally are less likely to use cannabis and e-cigarettes, the findings also revealed that some of them (e.g., younger individuals, those with anxiety/depression, lower education, identified as a man, or used alcohol) are more likely to use these substances.

While our study provides valuable insights into the disparities in e-cigarette and cannabis use between U.S.-born and immigrants, it is not without limitations. This study was based on web survey, which excludes individuals without internet access, with limited comprehension of the study materials, and does not allow to determine if the qualified participants complete the online survey themselves; this often result in disproportionate distributions among groups leading to under or overestimation of findings. The study’s cross-sectional design limits the ability to establish causal relationships between immigration status, mental health disorder symptoms, and substance use behaviors. The reliance on self-reported data may also introduce response bias. Also, the study did not assess the severity of e-cigarette or cannabis use regarding mental health because only patterns of dual use frequency were assessed between the immigrant and U.S.-born groups. This might have provided a more in-depth assessment of the differences between immigrant and U.S.-born groups regarding the association between anxiety/depression and frequency of use (exclusive and dual). Furthermore, the study did not account for the composition and potency of cannabis use, which may refer to a range of forms including combustibles, vapes, edibles, and flowers. These may differ considerably between immigrant and U.S.-born populations regarding the association with mental health factors. Due to limited samples across e-cigarette and cannabis use categories within immigrant and U.S.-born groups, we dichotomized alcohol use instead of assessing alcohol use frequency (1= not at all, 2= once during the month, 3= several times during the month, 4= once a week, 5= several times a week, 6= every day or almost every day, and 7= several times a day) in the past month. Assessing severity (frequency) of alcohol use in the past month might have provided greater differences between the U.S.-born and immigrant groups in terms of risk for cannabis and e-cigarette use. Because the study was conducted in only English language, the findings could not be generalized to non-English speaking, reading, and writing individuals. Only English language might have impacted the findings relating to factors such as loneliness which, in this population, was not associated with e-cigarette and cannabis use by immigration status. English language is a major barrier to communication and healthcare utilization among immigrants who come from culturally, ethnically, and linguistically diverse countries. Although we controlled for several factors in this study, residual confounders such as acculturation, generational status, country of origin, religion, access to healthcare services, and using other substances (e.g., cocaine, opioids, and ecstasy) could influence the observed associations. These residual confounders could have overestimated or underestimated the findings. Future research should address these limitations to provide a more comprehensive understanding of the factors influencing e-cigarette and cannabis use among U.S.-born and immigrant populations in the United States.

## 5. Conclusions

Our study contributes to expanding the limited health disparity literature on the prevalence of e-cigarette and cannabis use and its association with mental health disorder symptoms based on immigration status. The findings revealed significant mental health risks for e-cigarette, cannabis, and their dual use among immigrants and U.S.-born, especially among U.S.-born. However, these associations varied depending on specific substance use behavior and mental health disorder symptoms across immigrant and U.S.-born populations. Exclusive e-cigarette use behavior was not associated with anxiety/depression symptoms among U.S.-born, but it was associated with anxiety/depression symptoms among immigrants. Anxiety/depression symptoms, particularly the severe symptoms, were associated with higher likelihoods of exclusive cannabis use and dual use among immigrants and U.S.-born. The general effect sizes of dual-use associated with anxiety/depression symptoms were higher among U.S.-born, but the effect sizes of exclusive e-cigarette and cannabis use associated with anxiety/depression symptoms were higher among immigrants. Exclusive e-cigarette and dual use behaviors were not associated with PTSD symptoms across immigrant and U.S.-born individuals, while exclusive cannabis use behavior was associated with PTSD symptoms among immigrant and U.S.-born individuals. Exclusive and dual use of e-cigarettes and cannabis was not associated with loneliness across immigrant and U.S.-born individuals. The findings suggest the need to disaggregate data to examine specific substance use behaviors and mental health disorder symptoms to improve personalized substance use and mental health interventions in addressing health disparities, especially in minority populations. Future longitudinal or prospective studies should explore the mechanisms driving the associations between substance use behavior and mental health, especially the immigration status-related disparities.

## Figures and Tables

**Table 1:** Descriptive and bivariate analyses of the past-month exclusive e-cigarette use, cannabis use, and their dual use by sociodemographic characteristics, mental health disorder symptoms, and alcohol use among U.S.-born (n= 3672) and immigrants (n= 1094).

	U.S.-born						Immigrants					
	Overall sample	None used	Exclusive e-cigarette use	Exclusive cannabis use	Dual use of e-cigarettes and cannabis		Overall sample	None used	Exclusive e-cigarette use	Exclusive cannabis use	Dual use of e-cigarettes and cannabis	
	
	N (%)	n (%)	n (%)	n (%)	n (%)	p-value	N (%)	n (%)	n (%)	n (%)	n (%)	p-value
	
**Overall**		2371 (64.57)	261 (7.11)	497 (13.53)	543 (14.79)			880 (80.44)	69 (6.31)	55 (5.03)	90 (8.23)	

**Age groups**						<0.001						<0.001
18–25	449 (12.23)	236 (52.56)	38 (8.46)	68 (15.14)	107 (23.83)		232 (21.21)	172 (74.14)	22 (9.48)	20 (8.62)	18 (7.76)	
26–34	734 (19.99)	392 (53.41)	46 (6.27)	126 (17.17)	170 (23.16)		201 (18.37)	143 (71.14)	13 (6.47)	14 (6.97)	31 (15.42)	
35–49	1186 (32.30)	697 (58.77)	113 (9.53)	155 (13.07)	221 (18.63)		320 (29.25)	261 (81.56)	18 (5.62)	12 (3.75)	29 (9.06)	
50–64	957 (26.06)	734 (76.70)	56 (5.85)	125 (13.06)	42 (4.39)		203 (18.56)	173 (85.22)	13 (6.40)	7 (3.45)	10 (4.93)	
≥65	346 (9.42)	312 (90.17)	8 (2.31)	23 (6.65)	3 (0.87)		138 (12.61)	131 (94.93)	3 (2.17)	2 (1.45)	2 (1.45)	
**Gender identity**						<0.001						<0.001
Man	1317 (35.87)	765 (58.09)	102 (7.74)	171 (12.98)	279 (21.18)		362 (33.09)	269 (74.31)	26 (7.18)	18 (4.97)	49 (13.54)	
Woman	2313 (62.99)	1581 (68.35)	157 (6.79)	322 (13.92)	253 (10.94)		696 (63.62)	592 (85.06)	39 (5.60)	30 (4.31)	35 (5.03)	
Non-binary/transgender/other	42 (1.14)	25 (59.52)	2 (4.76)	4 (9.52)	11 (26.19)		36 (3.29)	19 (52.78)	4 (11.11)	7 (19.44)	6 (16.67)	
**Sexual orientation**						<0.001						<0.001
Heterosexual	3297 (89.79)	2179 (66.09)	236 (7.16)	418 (12.68)	464 (14.07)		960 (87.75)	791 (82.40)	58 (6.04)	38 (3.96)	73 (7.60)	
Sexual minority	375 (10.21)	192 (51.20)	25 (6.67)	79 (21.07)	79 (21.07)		134 (12.25)	89 (66.42)	11 (8.21)	17 (12.69)	17 (12.69)	
**Race/ethnicity**						<0.001						<0.001
Latino/Hispanic	503 (13.70)	313 (62.23)	32 (6.36)	71 (14.12)	87 (17.30)		387 (35.37)	299 (77.26)	33 (8.53)	20 (5.17)	35 (9.04)	
Black/African American	983 (26.77)	573 (58.29)	58 (5.90)	165 (16.79)	187 (19.02)		200 (18.28)	153 (76.50)	6 (3.00)	15 (7.50)	26 (13.00)	
White	1818 (49.51)	1229 (67.60)	149 (8.20)	228 (12.54)	212 (11.66)		177 (16.18)	138 (77.97)	16 (9.04)	10 (5.65)	13 (7.34)	
Other	368 (10.02)	256 (69.57)	22 (5.98)	33 (8.97)	57 (15.49)		330 (30.16)	290 (87.88)	14 (4.24)	10 (3.03)	16 (4.85)	
**Level of education**						<0.001						0.006
Less than High School	180 (4.90)	93 (51.67)	9 (5.00)	32 (17.78)	46 (25.56)		74 (6.76)	48 (64.86)	10 (13.51)	5 (6.76)	11 (14.86)	
High School diploma or GED	903 (24.59)	560 (62.02)	78 (8.64)	143 (15.84)	122 (13.51)		196 (17.92)	156 (79.59)	14 (7.14)	11 (5.61)	15 (7.65)	
Some college/vocational or technical school	1314 (35.78)	846 (64.38)	85 (6.47)	220 (16.74)	163 (12.40)		264 (24.13)	205 (77.65)	20 (7.58)	19 (7.20)	20 (7.58)	
College or higher degree	1275 (34.72)	872 (68.39)	89 (6.98)	102 (8.00)	212 (16.63)		560 (51.19)	471 (84.11)	25 (4.46)	20 (3.57)	44 (7.86)	
**US Census Region**						0.002						0.781
West	723 (19.69)	436 (60.30)	46 (6.36)	109 (15.08)	132 (18.26)		293 (26.78)	228 (77.82)	18 (6.14)	17 (5.80)	30 (10.24)	
Midwest	694 (18.90)	446 (64.27)	45 (6.48)	104 (14.99)	99 (14.27)		117 (10.69)	99 (84.62)	6 (5.13)	3 (2.56)	9 (7.69)	
Northeast	560 (15.25)	385 (68.75)	28 (5.00)	80 (14.29)	67 (11.96)		214 (19.56)	177 (82.71)	14 (6.54)	9 (4.21)	14 (6.54)	
South	1695 (46.16)	1104 (65.13)	142 (8.38)	204 (12.04)	245 (14.45)		470 (42.96)	376 (80.00)	31 (6.60)	26 (5.53)	37 (7.87)	
**Anxiety/depression symptoms**						<0.001						<0.001
Negative/Normal	1848 (50.33)	1416 (76.62)	128 (6.93)	183 (9.90)	121 (6.55)		639 (58.41)	556 (87.01)	34 (5.32)	19 (2.97)	30 (4.69)	
Mild	840 (22.88)	521 (62.02)	62 (7.38)	128 (15.24)	129 (15.36)		235 (21.48)	185 (78.72)	14 (5.96)	15 (6.38)	21 (8.94)	
Moderate	513 (13.97)	234 (45.61)	37 (7.21)	91 (17.74)	151 (29.43)		122 (11.15)	81 (66.39)	11 (9.02)	11 (9.02)	19 (15.57)	
Severe	471 (12.83)	200 (42.46)	34 (7.22)	95 (20.17)	142 (30.15)		98 (8.96)	58 (59.18)	10 (10.20)	10 (10.20)	20 (20.41)	
**PTSD status**						<0.001						<0.001
Met criteria but no PTSD	844 (22.98)	487 (57.70)	58 (6.87)	149 (17.65)	150 (17.77)		208 (19.01)	168 (80.77)	10 (4.81)	7 (3.37)	23 (11.06)	
Did not meet criteria	2450 (66.72)	1705 (69.59)	180 (7.35)	263 (10.73)	302 (12.33)		795 (72.67)	656 (82.52)	49 (6.16)	31 (3.90)	59 (7.42)	
Met criteria and had PTSD	378 (10.29)	179 (47.35)	23 (6.08)	85 (22.49)	91 (24.07)		91 (8.32)	56 (61.54)	10 (10.99)	17 (18.68)	8 (8.79)	
**Loneliness score (M [SD])**	5.06 (2.06)	4.76 (2.01)	5.08 (2.00)	5.54 (2.13)	5.92 (1.92)	<0.001	4.79 (1.93)	4.66 (1.88)	4.75 (1.85)	5.58 (2.16)	5.67 (2.01)	<0.001
**Past-month alcohol use**						<0.001						<0.001
No use	1457 (39.68)	1212 (83.18)	63 (4.32)	125 (8.58)	57 (3.91)		528 (48.26)	487 (92.23)	21 (3.98)	12 (2.27)	8 (1.52)	
Used	2215 (60.32)	1159 (52.33)	198 (8.94)	372 (16.79)	486 (21.94)		566 (51.74)	393 (69.43)	48 (8.48)	43 (7.60)	82 (14.49)	

Statistical significance at p<0.05. All p-values are based on chi-square tests for the categorical variables and ANOVA tests for the continuous variables.

**Table 2.1:** Multinomial logistic regression analysis of past-month exclusive e-cigarette use, cannabis use, dual use, and their associations with immigration status, adjusting for sociodemographic characteristics, mental health symptoms, and alcohol use among adults living in the U.S. (N= 4766).

	Base/reference category: None used		

	Exclusive e-cigarette use	Exclusive cannabis use	Dual use of e-cigarettes and cannabis
	
	RRR (95% CI)	RRR (95% CI)	RRR (95% CI)
**Immigration status**			
U.S.-born	Ref	Ref	Ref
Immigrants	0.85 (0.62, 1.17)	0.37*** (0.27, 0.51)	0.46*** (0.35, 0.62)

**Table 2.2:** Multinomial logistic regression analysis of past-month exclusive e-cigarette use, cannabis use, dual use, and their associations with sociodemographic characteristics, mental health disorder symptoms, and alcohol use among U.S.-born (n= 3672) and immigrants (n= 1094).

	U.S.-born			Immigrants		
	Base/reference category: None used Base/reference category: None used					
	Exclusive e-cigarette use	Exclusive cannabis use	Dual use of e-cigarettes and cannabis	Exclusive e-cigarette use	Exclusive cannabis use	Dual use of e-cigarettes and cannabis
	
	RRR (95% CI)	RRR (95% CI)	RRR (95% CI)	RRR (95% CI)	RRR (95% CI)	RRR (95% CI)
**Age groups**						
18–25	Ref	Ref	Ref	Ref	Ref	Ref
26–34	0.64 (0.40, 1.02)	1.11 (0.77, 1.59)	0.79 (0.56, 1.12)	0.79 (0.36, 1.76)	1.12 (0.48, 2.58)	1.83 (0.86, 3.93)
35–49	0.89 (0.58, 1.36)	0.94 (0.66, 1.34)	0.74 (0.53, 1.04)	0.57 (0.27, 1.20)	0.49 (0.21, 1.16)	0.95 (0.44, 2.03)
50–64	0.42*** **(0.26, 0.67)**	0.75 (0.52, 1.09)	0.17*** **(0.11, 0.27)**	0.59 (0.27, 1.33)	0.48 (0.18, 1.30)	0.55 (0.22, 1.38)
≥65	0.16*** **(0.07, 0.36)**	0.40** **(0.23, 0.69)**	0.03*** **(0.01, 0.10)**	0.14** **(0.04, 0.52)**	0.18* **(0.03, 0.88)**	0.12* **(0.02, 0.62)**
**Gender identity**						
Man	Ref	Ref	Ref	Ref	Ref	Ref
Woman	0.69** **(0.52, 0.91)**	0.70** **(0.56, 0.88)**	0.34*** **(0.27, 0.42)**	0.58 (0.33, 1.02)	0.50* **(0.26, 0.99)**	0.23*** **(0.13, 0.40)**
Non-binary/transgender/other	0.46 (0.10, 2.08)	0.30* **(0.10, 0.93)**	0.58 (0.24, 1.41)	1.61 (0.39, 6.58)	1.85 (0.51, 6.67)	0.58 (0.16, 2.16)
**Sexual orientation**						
Heterosexual	Ref	Ref	Ref	Ref	Ref	Ref
Sexual minority	1.06 (0.67, 1.69)	1.63** **(1.19, 2.24)**	1.25 (0.89, 1.76)	0.91 (0.39, 2.15)	2.08 (0.92, 4.69)	1.59 (0.75, 3.37)
**Race/ethnicity**						
Latino/Hispanic	0.66 (0.43, 1.01)	0.95 (0.69, 1.31)	1.00 (0.72, 1.40)	0.65 (0.33, 1.30)	0.61 (0.25, 1.46)	0.77 (0.35, 1.69)
Black/African American	0.75 (0.53, 1.05)	1.59*** **(1.24, 2.05)**	1.72*** **(1.31, 2.26)**	0.25** **(0.09, 0.69)**	0.96 (0.38, 2.45)	1.32 (0.57, 3.05)
White	Ref	Ref	Ref	Ref	Ref	Ref
Other	0.89 (0.54, 1.45)	0.73 (0.48, 1.10)	1.32 (0.89, 1.97)	0.41* **(0.18, 0.2)**	0.52 (0.19, 1.41)	0.55 (0.23, 1.32)
**Level of education**						
Less than High School	Ref	Ref	Ref	Ref	Ref	Ref
High School diploma or GED	1.46 (0.69, 3.06)	0.72 (0.45, 1.15)	0.49** **(0.30, 0.80)**	0.49 (0.19, 1.26)	0.75 (0.22, 2.53)	0.65 (0.23, 1.85)
Some college/vocational or technical school	0.95 (0.45, 1.99)	0.64 (0.41, 1.03)	0.38*** **(0.24, 0.62)**	0.53 (0.21, 1.33)	1.22 (0.38, 3.92)	0.62 (0.23, 1.69)
College or higher degree	0.82 (0.39, 1.74)	0.28*** **(0.17, 0.46)**	0.43** **(0.26, 0.70)**	0.26** **(0.11, 0.64)**	0.55 (0.17, 1.73)	0.48 (0.19, 1.22)
**US Census Region**						
West	Ref	Ref	Ref	Ref	Ref	Ref
Midwest	0.87 (0.56, 1.37)	0.72* **(0.52, 0.99)**	0.59** **(0.42, 0.84)**	0.80 (0.29, 2.20)	0.31 (0.08, 1.19)	0.68 (0.27, 1.73)
Northeast	0.69 (0.42, 1.14)	0.71 (0.50, 1.01)	0.50*** **(0.34, 0.73)**	1.14 (0.53, 2.46)	0.81 (0.33, 2.00)	0.84 (0.39, 1.83)
South	1.21 (0.83, 1.74)	0.54*** **(0.40, 0.72)**	0.58*** **(0.43, 0.77)**	1.03 (0.54, 1.96)	0.79 (0.39, 1.61)	0.82 (0.45, 1.51)
**Anxiety/depression symptoms**						
Negative/Normal	Ref	Ref	Ref	Ref	Ref	Ref
Mild	1.18 (0.82, 1.69)	1.46* **(1.10, 1.94)**	2.37*** **(1.72, 3.28)**	1.27 (0.62, 2.61)	1.68 (0.73, 3.87)	1.94 (0.96, 3.91)
Moderate	1.47 (0.93, 2.32)	2.11*** **(1.49, 2.99)**	5.39*** **(3.77, 7.73)**	2.55* **(1.07, 6.08)**	2.65 (1.00, 7.01)	4.70*** **(2.08, 10.61)**
Severe	1.64 (0.97, 2.75)	2.45*** **(1.66, 3.61)**	6.77*** **(4.48, 10.23)**	3.22* **(1.21, 8.60)**	3.09* **(1.02, 9.32)**	6.88*** **(2.78, 17.07)**
**PTSD status**						
Met criteria but no PTSD	Ref	Ref	Ref	Ref	Ref	Ref
Did not meet criteria	0.95 (0.68, 1.32)	0.62*** **(0.49, 0.79)**	0.81 (0.62, 1.06)	1.39 (0.66, 2.88)	1.54 (0.63, 3.74)	0.84 (0.46, 1.55)
Met criteria and had PTSD	0.87 (0.50, 1.11)	1.21 (0.85, 1.73)	1.09 (0.75, 1.60)	2.13 (0.77, 5.1)	4.47** **(1.59, 12.59)**	0.59 (0.22, 1.58)
**Loneliness score**	1.02 (0.94, 1.11)	1.02 (0.96, 1.08)	1.04 (0.97, 1.11)	0.85 (0.71, 1.01)	0.97 (0.81, 1.18)	1.06 (0.90, 1.24)
**Past-month alcohol use**						
No use	Ref	Ref	Ref	Ref	Ref	Ref
Used	3.60*** **(2.65, 4.89)**	3.52*** **(2.79, 4.44)**	10.13*** **(7.41, 13.84)**	3.70*** **(2.06, 6.64)**	6.49*** **(3.09, 13.63)**	15.37*** **(6.92, 34.17)**

RRR = Relative risk ratio. 95% CI = 95% confidence interval.

Statistical significance at *p<0.05

** p<0.01

*** p<0.001.

Ref= reference.
